# Outcome of breast cancer screening in Denmark

**DOI:** 10.1186/s12885-017-3929-6

**Published:** 2017-12-28

**Authors:** Elsebeth Lynge, Martin Bak, My von Euler-Chelpin, Niels Kroman, Anders Lernevall, Nikolaj Borg Mogensen, Walter Schwartz, Adam Jan Wronecki, Ilse Vejborg

**Affiliations:** 1 Department of Public Health, University of Copenhagen, Øster Farimagsgade 5, 1014 Copenhagen, Denmark; 2 Department of Pathology, Odense University Hospital, J. B. Winsløws Vej 15, 5000 Odense, Denmark; 3 Department of Public Health, University of Copenhagen, Øster Farimagsgade 5, 1014 Copenhagen, Denmark; 4 Department of Breast Surgery, Copenhagen University Hospital Herlev, Blegdamsvej 9, 2100 Copenhagen, Denmark; 5 Department of Public Health Programmes, Randers Regional Hospital, Skovlyvej 15, 8930 Randers NØ, Denmark; 6 Radiology Department, Ringsted Hospital, Bøllingsvej 30, 4100 Ringsted, Denmark; 7 Mammography Centre, Odense University Hospital, J. B. Winsløws Vej 15, 5000 Odense, Denmark; 8 Radiology Department, Aalborg Univeristy Hospital, Hobrovej 18-22, 9000 Aalborg, Denmark; 9 Radiology Department, Copenhagen University Hospital Rigshospitalet, Blegdamsvej 9, 2200 Copenhagen, Denmark

**Keywords:** Breast cancer, Ductal carcinoma in situ., Screening., Mammography.

## Abstract

**Background:**

In Denmark, national roll-out of a population-based, screening mammography program took place in 2007–2010. We report on outcome of the first four biennial invitation rounds.

**Methods:**

Data on screening outcome were retrieved from the 2015 and 2016 national screening quality reports. We calculated coverage by examination; participation after invitation; detection-, interval cancer- and false-positive rates; cancer characteristics; sensitivity and specificity, for Denmark and for the five regions.

**Results:**

At the national level coverage by examination remained at 75–77%; lower in the Capital Region than in the rest of Denmrk. Detection rate was slightly below 1% at first screen, 0.6% at subsequent screens, and one region had some fluctuation over time. Ductal carcinoma in situ (DCIS) constituted 13–14% of screen-detected cancers. In subsequent rounds, 80% of screen-detected invasive cancers were node negative and 40% ≤10 mm. False-positive rate was around 2%; higher for North Denmark Region than for the rest of Denmark. Three out of 10 breast cancers in screened women were diagnosed as interval cancers.

**Conclusions:**

High coverage by examination and low interval cancer rate are required for screening to decrease breast cancer mortality. Two pioneer local screening programs starting in the 1990s were followed by a decrease in breast cancer mortality of 22–25%. Coverage by examination and interval cancer rate of the national program were on the favorable side of values from the pioneer programs. It appears that the implementation of a national screening program in Denmark has been successful, though regional variations need further evaluation to assure optimization of the program.

**Electronic supplementary material:**

The online version of this article (10.1186/s12885-017-3929-6) contains supplementary material, which is available to authorized users.

## Background

Breast cancer has been the most common cancer disease in Danish women ever since national cancer registration started in 1943. However, the disease has been on a steady increase with a doubling of the age-standardised rate (Nordic standard population) from 69 per 100,000 in the early 1940s to 145 today [1]. This development is not surprising, given that the risk of breast cancer is closely related to the woman’s reproductive history. Women born in 1929–1947 reported an average age of menarche at 13.56 years [2], while this had decreased to 13.30 years for women born primarily in 1960–1980 [3]. At the same time, the age at first birth has increased, it was 23 years around 1960 and 29 years in 2015 [4]. The proportion of obese women increased from 1994 to 2010 [5]. With this considerable extension of the time window from menarche to first birth and with increased obesity, Danish women became more vulnerable to breast cancer, and primary prevention is difficult.

Since the late 1970s, node negative and moderately node positive breast cancers dominated the increasing incidence, probably as a result of emerging breast awareness [6]. Furthermore, new treatment modalities in the form of staging with axillary lymph node dissection; and hormonal and adjuvant chemotherapy treatment have helped to keep breast cancer mortality in control. Breast cancer mortality was at the level of 45 per 100,000 in the early 1940s and only increased to 51 per 100,000 in the mid 1990s, where after a decrease has been observed to 33 per 100,000 in 2014 [1]. However, breast cancer still causes 1100 deaths per year; being the second cause of cancer death in Danish women.

In the 1980s, a number of randomized controlled trial – first of all from Sweden – showed that screening mammography with early detection of small breast cancers could help to reduce breast cancer mortality [7]. In 2003, the European Union recommended population-based breast cancer screening [8]. In Denmark, this development led to the start of some regional screening programs in the early 1990s, and to national roll-out of screening in 2007–2010.

The long-tem purpose of breast cancer screening is to reduce breast cancer mortality [9]. When a screening program is implemented, it is, however, necessary to know relatively quickly whether or not the program is on the right track. The randomized controlled trials showed that short-term indicators of the screening program like the interval cancer rate, rate of screen-detected cancers, etc. correlated well with the later decline in breast cancer mortality [10]. It has therefore become standard to evaluate the early outcome of a screening program based on a set of short term indicators [11]. We report here on the short-term outcomes of the national Danish screening program.

## Methods

### Breast cancer screening program

In the 1990s, Denmark was divided into 16 administrative areas. A population-based screening program started in one of these areas; the municipality of Copenhagen; in April 1991 [12]. This was followed by programs in the county of Funen in November 1993 [13], and in the municipality of Frederiksberg in June 1994. Women aged 50–69 years were personally invited to biennial screening at dedicated screening clinics being stationary or mobile. One invitation, eventually followed by two reminders or another invitation, were sent to all women unless they had informed the program that they did not want to be invited. Furthermore women terminally ill, in breast cancer treatment/control, or with mammography within the last 12 months were not invited if this information was known to the screening program.

Trained radiographers took the mammograms, and screening did not include clinical breast examination. Two-view mammography was used at the first examination, and during the first ten years of the programs, women with fatty breast tissue would be scheduled for one-view at next screen, whereas women with mixed/dense tissue would be scheduled for two-view mammography. From around 2001, two-view mammography was used in all examinations. Mammograms were read independently by two trained radiologists. Women with suspicious finding were recalled for diagnostics at the hospital radiology departments. In 2006, the programs switched from analog to digital mammography.

In 1999, it became mandatory for Danish counties to offer breast cancer screening, but it was up to the minister of health to decide on the time of implementation of this law [14]. Screening started in the small county of Bornholm in 2001, and in Zealand county in 2004. In 2005, the breast cancer screening program in the municipality of Copenhagen was reported to have been followed by a 25% reduction in breast cancer mortality in the target population and a 37% reduction amongst participants [15]. Based on these results, the minister of health required that the new regions (from 1 January 2007) should start breast cancer screening before the end of 2007 and that the rollout should be completed at the end of 2009 [16]. A national quality database was implemented, and a steering committee was appointed with the responsibilities to monitor quality and to draw up national clinical guidelines for the screening [17]. The national screening program was in all aspects organized similarly to the pioneer programs.

### Danish quality assurance Data Base on mammography screening – DKMS

The breast cancer screening program is monitored annually based on 11 quality assurance indicators [11] inspired by the European guidelines [18]: radiation dose; participation after invitation; adherence to screening intervals; recall; interval cancers; invasive cancers as proportion of screen-detected cancers (invasive + ductal carcinoma in situ (DCIS)); node negative as proportion of invasive cancers; invasive cancers ≤10 mm as proportion of invasive cancers; benign vs. malignant operations; and response time. Using a slightly modified version of these indicators, we report here on: coverage by examination; participation after invitation; detection rate; interval cancer rate; and cancer characteristics (proportion invasive, node-negative, and size). From the data we furthermore calculated number of false positive screens; sensitivity; and specificity. The reported data covered the first four approximately biennial invitation rounds. Interval cancer data were not available for the fourth round.

Data in the DKMS on the target population for screening are retrieved from the Central Population Register (CPR) including all persons with a permanent address at any time since 1968. Data on invitations to screening are retrieved from the regional booking systems, which are based on always updated versions of CPR. Data on participation in screening and on screening outcome are retrieved from the Danish Patient Register. This register includes information on all out-and inpatient contacts to Danish hospitals. As all screening, assessment of women with suspicious screens, and eventual treatment take place in hospitals, all contacts related to the screening program will be recorded in the Danish Patient Register. Data on screen-detected and interval breast cancers are retrieved from the Danish Pathology Register (Patobank), which records data on all pathology specimens from Denmark. Opportunistic screening is rare in Denmark [19], and data on mammography outside the program are not included in the DKMS. Annual DKMS reports have been published since 2010. However, due to updates and correction of data files reported numbers vary somewhat from one report to another. We have used data from the reports from 2015 [20] and 2016 [21], respectively. The DKMS data are published without age-specification. This should, however, not affect the results, as the age-distribution for women aged 50–69 years varies for each 5-year age-group by only +/−1% across the five Danish regions [22].

### Statistics

The target population for screening is defined as women aged 50–69 years and living in a given region at the start of an invitation round; in practice these dates have been defined as 1 January 2008 for first, 2010 for second, 2012 for third, and 2014 for fourth round. Invited to screening are women aged 50–69 years defined in running age during the invitation round meaning that women start to be invited when they turn 50 years and end being invited when they turn 70 years. The open population of invited women could thus be larger than the target population. Some invitation rounds furthermore lasted longer than 24 months, Additional file 1: Table S1. However, in some rounds in the Southern Denmark and the Capital regions women who had not responded to three previous invitations or reminders were excluded from invitation in the following invitation round. Here we report coverage by examination calculated as number of screened women divided by the target population, and participation after invitation as number of screened women divided by the number of invited women.

Screened women with abnormal findings (positive screens) were referred for assessment, while women with normal findings (negative screens) were returned to routine screening. A screen-detected cancer was defined as a woman with a positive screen and diagnosed with invasive breast cancer or ductal carcinoma in situ (DCIS) within the next 6 months. Interval cancer was defined as invasive breast cancer diagnosed in women with negative screen within 24 months of the screening date (or before next screen), and in women with positive screen within 6–23 months of the sceening date. A false-positive screen was a women with a positive screen and no screen-detected cancer. The very small number of women with invasive breast cancer 6–23 months after a positive screen would be counted both as interval cancers and as false-positives, but it was not possible from the published data to separate out this very small group.

The screen-detection rate was: ((women with screen-detected invasive cancer + DCIS)/screened women). The false-positive rate: ((screen-positive women-(women with screen-detected invasive cancer + DCIS))/screened women). Interval cancer rate was: (Interval cancers/(interval cancers + screen-detected invasive cancer + DCIS)). Sensitivity was: (Screen-detected cancer/(Screen-detected cancer + interval cancers)). Specificity was: ((Screened women-(Screen-detected cancer + interval cancers)-(false-positive))/(Screened women-(Screen-detected cancer + interval cancers)). The cumulative false-positive risk after four rounds of screening as calculated as [1-(1-fp_1_)(1-fp_2_)(1-fp_3_)(1-fp_4_)]_,_ where fp_i_ was the false-positive rate in a given invitation round [23]. 95% confidence intervals (CI) were calculated under the assumption of a binomial distribution of the numerator.

### Ethics

All data in this paper were quoted from publicly available databases.

## Results

**Fig. 1:**
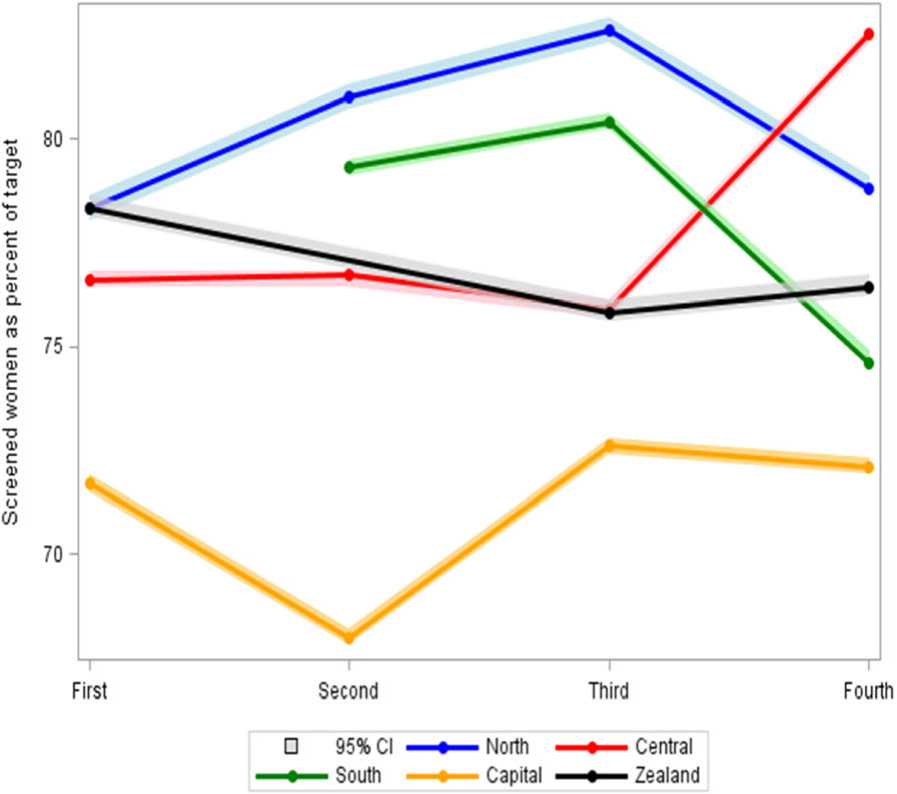
Coverage in screening mammography in Denmark 2008–2015 by invitation round and region. Percent and 95% confidence intervals. Notes: 1 South Denmark omitted in 1st round because only 70% of target population was invited. 2 Zealand omitted in 2nd round because the round was stopped before time to synchronize time periods across regions

Coverage by examination has remained stable at 75–77% throughout the first four rounds of the Danish screening mammography program, Table 1. While most of the regions had a coverage by examination fluctuating around the national average, the Capital Region was systematically below the average, being 68.0% (95% CI, 67.8–68.2) at its lowest in the second invitation round 2010–2011, Additional file 2: Table S2, Fig. 1. Participation after invitation was 77–84%.

**Table 1:** Overview of performance indicators in screening mammography in Denmark 2008–2015

Performance indicator	Invitation round			
	First 2008–2009%	Second 2010–2011%	Third 2012–2013%	Fourth 2014–2015%
Coverage of examination	75.4 (75.3–75.5)	75.0 (74.8–75.1)	76.7 (76.6–76.8)	76.4 (76.3–76.5)
Participation after invitation	76.4 (76.3–76.5)	81.8 (81.7–81.9)	84.3 (84.3–84.4)	82.1 (82.0–82.2)
Detection rate	0.93 (0.91–0.96)	0.62 (0.60–0.64)	0.67 (0.65–0.69)	0.61 (0.59–0.64)
False-positive rate	2.04 (2.00–2.08)	2.08 (2.04–2.12)	2.07 (2.03–2.11)	1.88 (1.84–1.92)
Invasive	87.5 (86.5–88.4)	86.3 (85.0–87.5)	86.4 (85.2–87.4)	85.8 (84.5–86.9)
DCIS	12.6 (11.6–13.5)	13.7 (12.5–15.0)	13.6 (12.6–14.8)	14.2 (13.1–15.5)
Lymph node neg	69.8 (68.4–71.2)	74.5 (72.8–76.2)	78.2 (76.7–79.6)	80.4 (78.8–81.8)
Small tumor	36.1 (34.4–37.8)	40.1 (38.2–42.1)	39.8 (38.0–41.5)	40.1 (38.3–42.0)
Interval cancer rate	17.9 (16.9–19.1)	28.9 (27.3–30.5)	26.3 (24.9–27.8)	NA
Sensitivity	82.1 (81.1–83.1)	71.2 (69.8–72.5)	73.7 (72.5–74.8)	NA
Specificity	97.9 (97.9–98.0)	97.9 (97.9–97.9)	97.9 (97.9–98.0)	NA

Notes:

*NA* not available

Percent and 95% confidence intervals

**Fig. 2:**
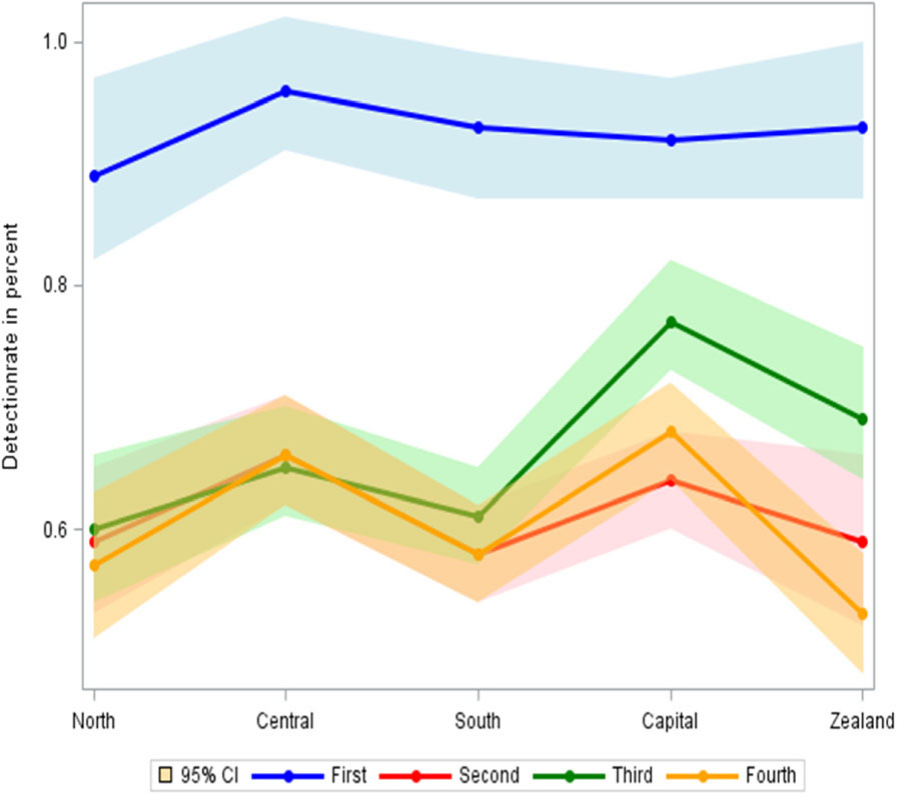
Detection rate in screening mammography in Denmark 2008–2015 by region and invitation round. Percent and 95% confidence intervals

The detection rate was 0.93% in the first invitation round which for the majority of screened women was the prevalence screen, and the detection rate fluctuated between 0.61 and 0.67% the the next three invitations rounds. There was limited regional difference in the detection rate during the first round. However, in the third round the Capital Region had a detection rate of 0.77% (95% CI, 0.73–0.82), and considerable variation was seen in the detection rate in Region Zealand from 0.69% in the third round to 0.53% (95% CI, 0.48–0.58) in the fourth round, Fig. 2. The proportion of DCIS out of all screen-detected cases was stable over time being 13% in the first invitation round and 14% in the fourth. The regional variations were limited apart from fluctuations over time the in the small North Denmark Region. At the national level, the false-positive rate was fairly stable varying from 1.88% (95% CI, 1.84–1.92) to 2.08% (95% CI, 2.04–2.12) between rounds, Table 1. Region North Denmark was, however, systematically above the other regions, with false-positive rates varying from 2.7% (95% CI, 2.6–2.9) to 3.2% (95% CI, 3.0–3.3), Additional file 3: Table S3.

At the national level, invasive breast cancer as proportion of screen-detected cancers remained at 86–87% throughout the four rounds, Table 1, but during the first round there was a variation from 96% (95% CI, 93–97) in North Denmark to 85% (95% CI, 83–87) in South Denmark; a variation that diminished over time, Additional file 3: Table S3. As expected, the proportions of lymph node negative and small cancers were lower in the first round than later; 70% (95% CI, 68–71) and 36% (95% CI, 34–39), respectively. These proportions had increased to 80% (95% CI, 79–82) and 40% (95% CI,38–42), respectively, in the fourth round, Additional file 4: Table S4.

**Fig. 3:**
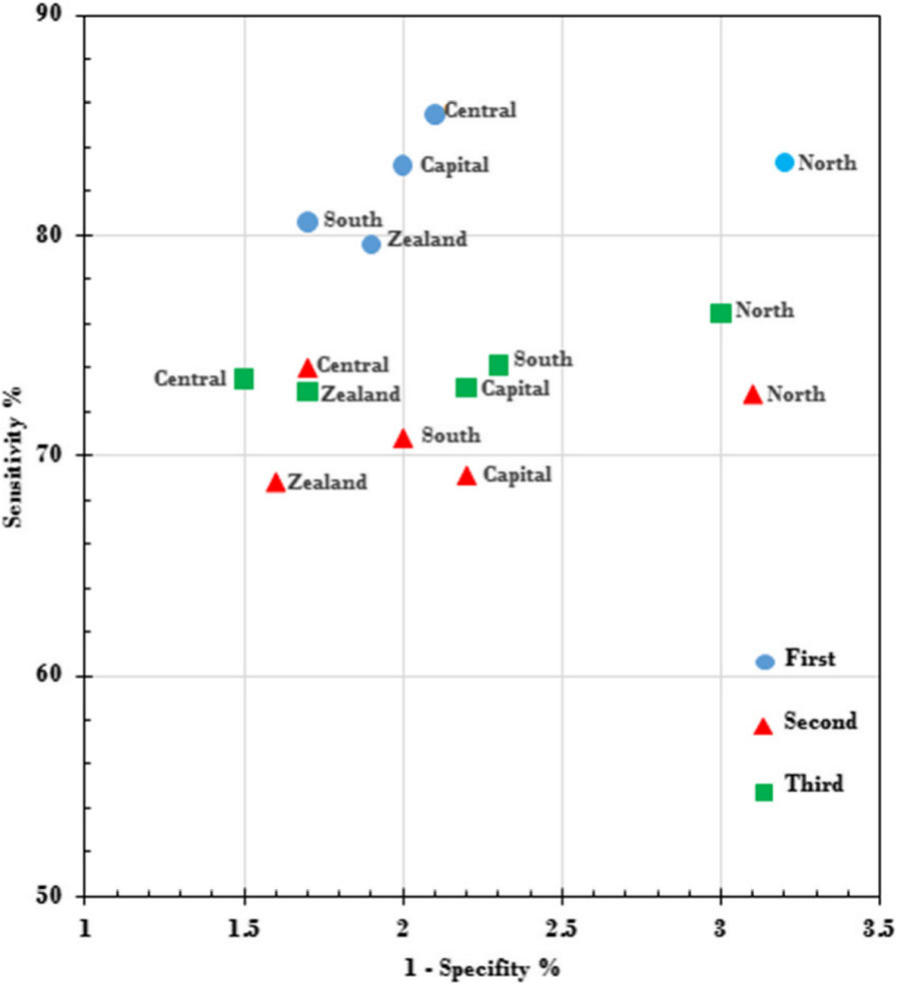
Sensitivity versus 1-specificity in screening mammography in Denmark 2015 by region and invitation round

As the first round was the prevalence screen for most women, the interval cancer rate at the national level was low, 18% (1032/(4724 + 1023), and the sensitivity was high, 82% (100–18%), Table 1. In the second and third rounds these numbers had changed to 26–29% and 71–74%, respectively. The specificity remained at 98% throughout the three rounds. There was, however, some variation across regions in sensitivity and specificity. The North Denmark Region had systematically lower specificity than the other regions. In the third invitation round, the specificity in the North Denmark Region was 97.0% (95% CI 96.9–97.1) as compared with 97.9% (95% CI 97.7–98.0) for all of Denmark, Additional file 5: Table S5. The outlier position of the North Denmark region is illustrated in Fig. 3.

## Discussion

### Main findings

Three quarters of Danish women followed the screening mammography program. Slightly below 1% of these women had a breast cancer (invasive or DCIS) detected at their first screen, and around 0.6% in subsequent screens. The proportion of DCIS remained constant 13–14% during the four invitation rounds; and in subsequent rounds 75–80% of the screen-detected invasive breast cancers were lymph node negative and 40% had a diameter equal to or below 10 mm. The screening program thus detected mainly invasive, lymph-node negative breast cancers with a high proportion of small cancers.

The false-positive rate remained around 2%, indicating that only a very small proportion of screened women underwent assessment without having breast cancer. On the other hand in subsequent rounds, a negative screen was no guarantee against breast cancer developing shortly after or having been overlooked in the first place. Three out of 10 breast cancers in screened women were diagnosed as interval cancers.

However, some regional differences were seen even in this population with only a total of 700,000 screen-targeted women. As seen in other urban settings [24], the coverage was relatively low in the Capital Region. This was in particular the case in the second round, where the coverage was 68% in the Capital Region as compared to the national average of 75%. The second round coincided with the publication of a study that claimed that screening lead to heavy overdiagnosis, and that one third of breasts were removed without reason [25]. Women in the Capital Region may have been more sensitive than other women to negative messages reported in the media. It is noteworthy that coverage in the Capital Region in the third and fourth rounds were back to the higher level from the first round.

The detection rate was surprisingly high in the Capital Region in the third round, 0.77% versus the national average of 0.67%. This was probably due a longer time interval between screens; the third invitation round lasted 28 months, and some women screened in the third round had skipped screening in the second round. In Region Zealand the detection rate fluctuated over time, in contrast to the situation in the other regions. Worrisome was the low detection rate of 0.53% in the third round where the national average was 0.61%. One possible explanation could be that the detection rate was high in the third round and that the prevalent pool of breast cancers was depleted at that time. But this should then have been followed by a high sensitivity which was not the case, Additional file 4: Table S4. The region has suffered from shortage of experienced radiologists. It remains to be seen what the sensitivity will be in Region Zealand after the fourth round.

The proportion of women with a false-positive screen was higher in the North Denmark Region than in Denmark on average; the cumulative risk being 11.4% as compared with the average of 7.9%. The region also had a conservative diagnostic practice, as both the detection rates and the proportion of DCIS were generally in the low end of the spectrum.

### Strengths and weaknesses

The DKMS data are nationwide and based on individually registered events. Several circumstances have, however, complicated the reporting. First, Denmark does not have a national invitation database. In order to follow the fate of an individual woman, data from the regional booking systems have to be linked with data from the Danish Patient Register and the Patobank; and these matches were not always perfect. It is though unlikely that this would have affected the results; e g. in the first invitation round a total of 509,932 women were screened and results were missing for only 88 of these women. Second, an invitation round should ideally have a length of 24 months. But due to lack of manpower this has not always been possible, and even the fourth round lasted 27 months in 3 of the 5 regions. This led to problems with allocation of data to the correct round; reflected in changes in numbers from one annual DKMS report to the next. For this reason only the latest updated data were quoted in this paper.

### Perspective for reduction in breast cancer mortality

Given the correlation observed in the randomised controlled trial between favourable outcomes of the short-term indicators and the later decline in breast cancer mortality [10], one may ask whether the Danish national program is on the right track. Here it might be reasonably to compare with the outcomes of the two pilot programs. There is, however, some confusion in the literature about the impact of these two pilot programs on breast cancer mortality and overdiagnosis. Before turning to a comparison with the present national program, it is therefore necessary to understand these seemingly contradictory results from the pilot programs.

The fact that two Danish administrative areas introduced breast cancer screening up to 17 years before the rest of Denmark constituted almost a “natural experiment”, and this has provided the basis for several evaluations of the effect of screening. Using individually linked cohort data from the Copenhagen program, Olsen et al. [15] found that breast cancer mortality in Copenhagen had decreased by 25% more than expected in the absence of screening; and Njor et al. [26] found a decrease of 22% for the Funen program. Using routine breast cancer mortality data from fixed age-groups, Jørgensen et al. [27] concluded that they “were unable to detect any effect of the Danish screening programmes on breast cancer mortality”.

There is, however, explanations for these seemingly contradictory results. First, the routine data used by Jørgensen et al. included breast cancer deaths from women diagnosed with breast cancer prior to the start of the screening program, and these women had no chance to benefit from screening. Olsen et al. used incidence-based mortality including only deaths from breast cancer in women diagnosed after the start of the screening program, and thus having had a chance to benefit from screening. Second, Jørgensen et al. looked only at average annual change in the trends of breast cancer mortality before and after start of screening. They left out observations from the first 7 years after start of the screening programs, and thus ignored changes in breast cancer mortality during this period. In fact, a recalculation of the data reported by Jørgensen et al. showed a decline of 13% in breast cancer mortality in the screening areas as compared with the decline in the non-screening areas [28]. Given that the Jørgensen et al. data were contaminated with breast cancer deaths in women diagnosed prior to screening, this 13% decline is fairly much in line with the 22–25% decline observed by Olsen et al. and Njor et al. in the non-contaminated data.

Overdiagnosis has been studied also based on the early Danish data. Using individually linked data from cohorts of women offered screening and followed for a minimum of 8 years after end of screening age, Njor et al. [29] estimated overdiagnosis to amount to 2.3%. Using routine data from fixed age-groups, Jørgensen et al. [25, 30] concluded that “1 in every 3 women aged 50 to 69 years diagnosed with breast cancer was overdiagnosed”. However, screening introduces a dynamic in the incidence of breast cancer with a prevalence peak, an artificial aging, and a compensatory dip [31, 32]. This dynamic is captured correctly only by following the cohorts of screened women. With the method used by Jørgensen et al., they were unable to capture the compensatory dip correctly. Jørgensen et al., furthermore measured differences instead of proportions, and thus inflated their estimate of overdiagnosis by geographical differences in breast cancer incidence prior to the introduction of screening [33].

The differences between the study approaches used by Olsen et al. and Njor et al. and the one used by Jørgensen et al. stress the superiority of using individually linked cohort data as opposed to routine statistics data in evaluation of screening outcomes. The most accurate estimate of the decline in breast cancer mortality in the pilot programs is therefore 25% for Copenhagen and 22% for Funen. The short-term indicators from the first four invitation rounds of the two Danish pilot programs in the municipality of Copenhagen ([12, 34] + unpublished material) and the county of Funen ([13, 34] + unpublished data) are summarized in Table 2.

**Table 2:** Coverage, interval cancer and false positive rates during the first four invitation rounds of the Danish pioneer screening mammography programs in the municipality of Copenhagen (1991–1998) and the county of Funen (1994–2001)

	Invitation round			
	First %	Second %	Third %	Fourth %
Copenhagen				
Coverage by examination	71 (70–71)	63 (63–64)	63 (62–63)	63 (63–64)
Interval cancer rate	14 (11–17)	28 (23–35)	27 (22–34)	33 (27–39)
False positive rate	5.5 (5.2–5.7)	3.9 (3.6–4.1)	2.5 (2.3–2.7)	2.4 (2.2–2.6)
Funen				
Coverage by examination	85 (84–85)	83 (83–84)	82 (82–83)	84 (84–84)
Interval cancer rate	18 (15–22)	34 (30–39)	39 (34–44)	32 (28–37)
False positive rate	1.7 (1.6–1.9)	1.1 (1.0–1.2)	1.1 (1.0–1.2)	1.0 (0.9–1.1)

Percent and 95% confidence intervals

In the randomised controlled trials a low interval cancer rate, a high screen-detection rate, a low proportion of stage II+ tumors, and a high proportion of small tumors were predictors of a later decline in breast cancer mortality [10]. At the population level one might add coverage by examination to this list of predictors. Copenhagen had a lower interval cancer rate than Funen during the first three rounds, which can probably explain why screened women in Copenhagen had a larger decrease in breast cancer mortality than screened women from Funen. Funen on the other had higher coverage by examination, and the two pioneer programs ended up with largely similar decreases in breast cancer mortality for screen-targeted women. It should be taken into account that breast cancer patients in Funen already prior to the implementation of screening had a better survival then breast cancer patients in the rest of Denmark [35], and that the Funen program deliberately aimed for a lower false positive rate than found in the start of the Copenhagen program, Table 2.

**Fig. 4:**
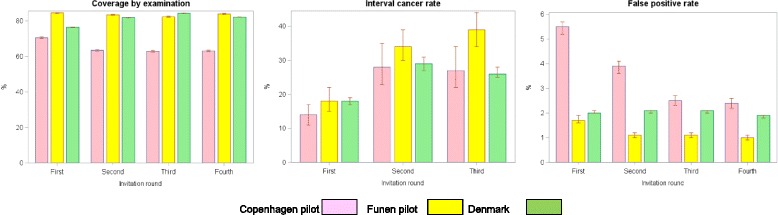
Coverage by examination, interval cancers rate and false positive rate in the first four invitation rounds of the pilot screening programs in Copenhagen and Funen and in the Danish national program. Percent and 95% confidence interval.

In the first four invitation rounds of the national program, the coverage by examination has been almost at the average of the coverage by examination in the pilot programs, Fig. 4. During the first three invitation rounds the interval cancer rate has been in line with the rate observed in the Copenhagen program. This means that the national program has both avoided the low coverage by examination in the pilot Copenhagen program and the high interval cancer rate in the pilot Funen program. On this basis one might expect that the national program will also result in a reduction in breast cancer mortality. Thorough cohort studies on incidence-based mortality are needed in order to investigate this. Women in the national program paid a price in terms of false-positive screens exceeding the low level in the pilot program in Funen.

Furthermore, as the majority of screen-detected breast cancers are node negative, Additional file 4: Table S4, women are spared axillary dissection. Given that 40% of screen detected tumors were ≤10 mm and that 80% were lymph node negative, a significant proportion of screen-detected tumors are expected to be low risk not in need of chemotherapy, but the DKMS data are too sparse on tumor biology to estimate the precise proportion.

## Conclusion

Fulfillment of short-term quality indicators is a prerequisite for a screening mammography program to achieve its purpose of reducing breast cancer mortality [10]. Our study showed that even within the small Danish population the variations in both screen-detection and false-positives rates were surprisingly large but all regions are working quite well in accordance with European and national guidelines. Screening mammography is a delicate balance between benefits and harms [36], and the Danish experiences illustrate the importance of close monitoring of short-term quality indicators.

## Additional files

Additional file 1: Table S1. Date of start and foreseen date of end of invitations rounds by region and length (in months) of invitation round. (DOCX 12 kb)

Additional file 2: Table S2. Number of women in target population, invited women and screened women by invitation round and region in screening mammography, Denmark 2008–2015. (DOCX 15 kb)

Additional file 3: Table S3. Number of screened women, recalled women, screen-detected breast cancers (incl. DCIS) and women with false positive screen by invitation round and region in screening mammography in Denmark, 2008–2015. (DOCX 15 kb)

Additional file 4: Table S4. Number of screen-detected cancers (Invasive + DCIS), screen-detected cancers (invasive only) and interval cancers (invasive only) by invitation round and region in screening mammography in Denmark 2008–2015. (DOCX 15 kb)

Additional file 5: Table S5. Number of screened women, screen-detected cancers (invasive + DCIS) interval cancers and women with false positive screens by invitations round and region in screening mammography in Denmark 2008–2015. (DOCX 15 kb)
