# Supplementary material for: Outcome of breast cancer screening in Denmark
Source: BMC Cancer. 2017 Dec 28;17:897. doi: 10.1186/s12885-017-3929-6 (PMC5745763; doi:10.1186/s12885-017-3929-6)
Supplement: Supplementary file 1 — Date of start and foreseen date of end of invitations rounds by region and length (in months) of invitation round. (DOCX 12 kb) [file 12885_2017_3929_MOESM1_ESM.docx]

Supplementary Table 1. Date of start and foreseen date of end of invitations rounds by region and length (in months) of invitation round

| Invitation  round |  | | | | |
| --- | --- | --- | --- | --- | --- |
|  | North | Central | South | Capital | Zealand |
| First: from | Medio 2007 | 22-02-2008 | 01-09-2007 | 20-12-2007 | 01-07-2008 |
| to | 15-08-2009 | 31-12-2009 | 16-07-2010 | 31-01-2010^1^ | 31-12-2010 |
| Months | 25.5 | 22 | 34.5 | 24.5 | 30 |
| Second: from | 16-08-2009 | 01-01-2010 | 09-08-2010 | 01-01-2010 | 01-01-2011 |
| to | 01-02-2012 | 31-12-2011 | 30-06-2012 | 31-12-2011 | 31-12-2011 |
| Months | 28.5 | 24 | 22 | 24 | 24 |
| Third: from | 01-07-2011 | 01-01-2012 | 01-07-2012 | 01-01-2012 | 01-01-2012 |
| to | 31-01-2014 | 31-12-2013 | 31-08-2014 | 30-04-2014 | 31-03-2014 |
| Months | 31 | 24 | 26 | 28 | 24 |
| Fourth: from | 15-10-2013^2^ | 01-01-2014 | 04-08-2014 | 01-05-2014 | 01-04-2014 |
| to | 31-12-2015 | 31-12-2015 | 06-08-2016 | 01-07-2016 | 31-07-2016 |
| Months | 26.5 | 24 | 24 | 27 | 27 |

Notes:

1. Bornholm: 31-03-2010
2. Varied between 15-10-2013 to 01-03-2014 for the three screening units
