# Supplementary material for: Outcome of breast cancer screening in Denmark
Source: BMC Cancer. 2017 Dec 28;17:897. doi: 10.1186/s12885-017-3929-6 (PMC5745763; doi:10.1186/s12885-017-3929-6)
Supplement: Supplementary file 2 — Number of women in target population, invited women and screened women by invitation round and region in screening mammography, Denmark 2008–2015. (DOCX 15 kb) [file 12885_2017_3929_MOESM2_ESM.docx]

Supplementary Table 2. Number of women in target population, invited women and screened women by invitation round and region in screening mammography, Denmark 2008-2015

|  | | North | Central | South | Capital | Zealand | DK |
| --- | --- | --- | --- | --- | --- | --- | --- |
| First | Target^1^ | 73,861 | 149,266 | 152,057 | 195,013 | 112,249 | 530,389^5^ |
|  | Invited^2^ | 74,851 | 149,292 | 118,490^4^ | 191,157 | 108,045 | 523,345^5^ |
|  | % invited | 101,3 | 100,0 | 77,9 | 98,0 | 96,3 | 98,7^5^ |
|  | Screened^2^ | 57,801 | 114,376 | 107,071^4^ | 139,750 | 87,934 | 399,861^5^ |
|  | % Target | 78.3 | 76.6 | 70.4 | 71.7 | 78.3 | 75.4^5^ |
|  | % Invited | 77.2 | 76.6 | 90.4 | 73.1 | 81.4 | 76.4^5^ |
| Second | Target^1^ | 75,461 | 153,391 | 155,202 | 197,898 | 114,394 | 581,952 |
|  | Invited^3^ | 74,825 | 144,188 | 128,493 | 185,656 | 48,557 | 533,162^7^ |
|  | % invited | 99,2 | 94,0 | 82,8 | 93,8 | -^6^ | - 91.6^7^ |
|  | Screened^3^ | 61,142 | 117,458 | 123,067 | 134,533 | 46,899 | 436,200^7^ |
|  | % Target | 81.0 | 76.6 | 79.3 | 68.0 | -^6^ | 75.0^7^ |
|  | % Invited | 81.7 | 81.5 | 95.8 | 72.5 | 96.6 | 81.8^7^ |
| Third | Target^1^ | 76,880 | 157,076 | 157,983 | 201,758 | 116,129 | 709,826 |
|  | Invited^3^ | 75,350 | 143,299 | 137,412 | 182,471 | 106,747 | 645,279 |
|  | % invited | 98,0 | 91,2 | 87,0 | 90,4 | 91,9 | - 90,9 |
|  | Screened^3^ | 63,490 | 119,180 | 127,006 | 146,454 | 88,050 | 544,180 |
|  | % Target | 82.6 | 75.9 | 80.4 | 72.6 | 75.8 | 76.7 |
|  | % Invited | 84.3 | 83.2 | 92.4 | 80.3 | 82.5 | 84.3 |
| Fourth | Target^1^ | 77,685 | 160,037 | 160,037 | 204,689 | 117,110 | 719,800 |
|  | Invited^3^ | 71,642 | 157,378 | 138,620 | 192,706 | 109,250 | 669,596 |
|  | % invited | 92,2 | 98,3 | 86,5 | 94,1 | 93,3 | - 93,0 |
|  | Screened^3^ | 61,248 | 132,035 | 119,490^8^ | 147,628^8^ | 89,510^8^ | 549,911^8^ |
|  | % Target | 78.8 | 82.5 | 74.6 | 72.1 | 76.4 | 76.4 |
|  | % Invited | 85.5 | 83.9 | 86.2 | 76.6 | 81.9 | 82.1 |

Notes:

1. Statistics Denmark, Statistikbanken: <http://www.statistikbanken.dk/statbank5a/default.asp?w=1680> [Accessed: 280617]
2. DKMS 2015, Table 2A
3. DKMS 2016, Tables 2A and B
4. Reported as 142,299 and 123,018, respectively, in DKMS 2010. But updated numbers from 2015 used here
5. Calculated without Southern Denmark
6. Interrupted invatition round, and coverage can therefore not be calculated
7. Calculated without Zealand
8. Slightly underreported due to lack of updated files
