# Supplementary material for: Outcome of breast cancer screening in Denmark
Source: BMC Cancer. 2017 Dec 28;17:897. doi: 10.1186/s12885-017-3929-6 (PMC5745763; doi:10.1186/s12885-017-3929-6)
Supplement: Supplementary file 3 — Number of screened women, recalled women, screen-detected breast cancers (incl. DCIS) and women with false positive screen by invitation round and region in screening mammography in Denmark, 2008–2015. (DOCX 15 kb) [file 12885_2017_3929_MOESM3_ESM.docx]

Suppelmentary Table 3. Number of screened women, recalled women, screen-detected breast cancers (incl. DCIS) and women with false positive screen by invitation round and region in screening mammography in Denmark, 2008-2015

|  | | North | Central | South | Capital | Zealand | DK |
| --- | --- | --- | --- | --- | --- | --- | --- |
| First | Screened^1,2^ | 57,757 | 114,375 | 107,030 | 139,750 | 87,932 | 506,844 |
|  | Recalled^2^ | 2,341 | 3,427 | 2,800 | 3,987 | 2,488 | 15,043 |
|  | BC+DCIS^3^ | 514 | 1,106 | 1,000 | 1,286 | 818 | 4,724 |
|  | BC Invasive^6^ | 491 | 981 | 852 | 1,102 | 705 | 4,131 |
|  | DCIS | 23 | 125 | 148 | 184 | 113 | 593 |
|  | DCIS/(BC+DCIS) | 4.5% | 11.3% | 14.8% | 14.3% | 13.8% | 12.6% |
|  | False pos. | 1,827 | 2,321 | 1,800 | 2,701 | 1,670 | 10,319 |
|  | Detection | 0.89 | 0.97 | 0.93 | 0.92 | 0.93 | 0.93 |
|  | False pos. % | 3.2 | 2.0 | 1.7 | 19 | 1.9 | 2.0 |
| Second | Screened^1,4^ | 61,141 | 117,455 | 123,067 | 133,740 | 46,898 | 482,301 |
|  | Recalled^4^ | 2,254 | 2,746 | 3,166 | 3,807 | 1,034 | 13,007 |
|  | BC+DCIS^5^ | 358 | 773 | 713 | 856 | 276 | 2,976 |
|  | BC Invasive^6^ | 320 | 682 | 603 | 732 | 226 | 2,563 |
|  | DCIS | 38 | 91 | 110 | 124 | 40 | 413 |
|  | DCIS/(BC+DCIS) | 10.6% | 11.8% | 15.4% | 14.5% | 14.5% | 13.8% |
|  | False pos. | 1,896 | 1,973 | 2,453 | 2,951 | 758 | 10,031 |
|  | Detection | 0.59 | 0.66 | 0.58 | 0.64 | 0.59 | 0.62 |
|  | False pos. % | 3.1 | 1.7 | 2.0 | 2.2 | 1.6 | 2.1 |
| Third | Screened^1,4^ | 63,490 | 119,179 | 126,999 | 144,330 | 88,050 | 542,048 |
|  | Recalled^4^ | 2,266 | 2,524 | 3,720 | 4,304 | 2,052 | 14,866 |
|  | BC+DCIS^5^ | 381 | 776 | 773 | 1,109 | 606 | 3,645 |
|  | BC Invasive^6^ | 348 | 668 | 654 | 956 | 524 | 3,150 |
|  | DCIS | 33 | 108 | 119 | 153 | 82 | 495 |
|  | DCIS/(BC+DCIS) | 9.4% | 13.9% | 15.4% | 13.8% | 13.5% | 13.6% |
|  | False pos. | 1,885 | 1,748 | 2,947 | 3,195 | 1,446 | 11,221 |
|  | Detection | 0.60 | 0.65 | 0.61 | 0.77 | 0.69 | 0.67 |
|  | False pos. % | 3.0 | 1.5 | 2.3 | 2.2 | 1.6 | 2.1 |
| Fourth | Screened^1,4^ | 61,248 | 132,035 | 119,419 | 145,298 | 89.508 | 547,508 |
|  | Recalled^4^ | 2,033 | 2,826 | 3,658 | 3,523 | 1,611 | 13,651 |
|  | BC+DCIS^5^ | 352 | 867 | 689 | 984 | 471 | 3,363 |
|  | BC Invasive^7^ | 300 | 717 | 593 | 858 | 416 | 2,884 |
|  | DCIS | 52 | 150 | 96 | 129 | 55 | 479 |
|  | DCIS/(BC+DCIS) | 14.8% | 17.3% | 13.9% | 13.1% | 11.7% | 14.2% |
|  | False pos. | 1,681 | 1,959 | 2,969 | 2,539 | 1,140 | 10,288 |
|  | Detection | 0.57 | 0.66 | 0.58 | 0.68 | 0.53 | 0.61 |
|  | False pos. % | 2.7 | 1.5 | 2.5 | 1.7 | 1.3 | 1.9 |

Notes:

1. Only screened women with a screening result
2. DKMS 2015, Table 4
3. DKMS 2015, Supplementary tables, Indicator 4, Table 2
4. DKMS 2016, Table 4
5. DKMS 2016, Supplementary tables, Indicator 6, Table 5
6. DKMS 2015, Table 6
7. DKMS 2016, Table 6
