# Supplementary material for: Outcome of breast cancer screening in Denmark
Source: BMC Cancer. 2017 Dec 28;17:897. doi: 10.1186/s12885-017-3929-6 (PMC5745763; doi:10.1186/s12885-017-3929-6)
Supplement: Supplementary file 4 — Number of screen-detected cancers (Invasive + DCIS), screen-detected cancers (invasive only) and interval cancers (invasive only) by invitation round and region in screening mammography in Denmark 2008–2015. (DOCX 15 kb) [file 12885_2017_3929_MOESM4_ESM.docx]

Supplementary Table 4. Number of screen-detected cancers (Invasive + DCIS), screen-detected cancers (invasive only) and interval cancers (invasive only) by invitation round and region in screening mammography in Denmark 2008-2015

|  | | North | Central | South | Capital | Zealand | DK |
| --- | --- | --- | --- | --- | --- | --- | --- |
| First | Screen.det (all)^1^ | 514 | 1,106 | 1,000 | 1,286 | 818 | 4,724 |
|  | Screen.det (inv)^2^ | 491 | 981 | 852 | 1,102 | 705 | 4,131 |
|  | % invasive | 96 | 89 | 85 | 86 | 86 | 87 |
|  | Lymph node info^3^ | 482 | 959 | 827 | 1,032 | 679 | 3,979 |
|  | Lymph node neg^3^ | 322 | 588 | 620 | 718 | 530 | 2778 |
|  | % neg | 67 | 61 | 75 | 70 | 78 | 70 |
|  | Size info^4^ | 252 | 630 | 772 | 748 | 686 | 3,088 |
|  | Size <10 mm^4^ | 70 | 217 | 284 | 288 | 256 | 1,115 |
|  | % <10 mm | 28 | 34 | 37 | 39 | 37 | 36 |
| Second | Screen.det (all)^5,8^ | 358 | 773 | 713 | 856 | 276 | 2,976 |
|  | Screen-det (inv)^5^ | 320 | 681 | 605 | 734 | 227 | 2,567 |
|  | % invasive | 89 | 88 | 85 | 86 | 82 | 86 |
|  | Lymph node info^6^ | 315 | 662 | 591 | 710 | 212 | 2,490 |
|  | Lymph node neg^6^ | 227 | 479 | 455 | 534 | 161 | 1,856 |
|  | % neg | 72 | 72 | 77 | 75 | 76 | 75 |
|  | Size info^7^ | 298 | 659 | 588 | 711 | 222 | 2,478 |
|  | Size <10 mm^7^ | 111 | 257 | 221 | 313 | 92 | 994 |
|  | % <10 mm | 37 | 39 | 38 | 44 | 41 | 40 |
| Third | Screen.det (all)^5,8^ | 381 | 776 | 773 | 1,109 | 606 | 3,645 |
|  | Screen.det (inv)^5^ | 348 | 663 | 656 | 957 | 524 | 3,148 |
|  | % invasive | 91 | 85 | 85 | 86 | 86 | 86 |
|  | Lymph node info^6^ | 337 | 647 | 638 | 920 | 504 | 3,046 |
|  | Lymph node neg^6^ | 242 | 516 | 501 | 719 | 403 | 2,381 |
|  | % neg | 72 | 80 | 79 | 78 | 80 | 78 |
|  | Size info^7^ | 323 | 635 | 633 | 939 | 516 | 3,046 |
|  | Size <10 mm^7^ | 142 | 245 | 254 | 356 | 214 | 1,211 |
|  | % <10 mm | 44 | 39 | 40 | 38 | 42 | 40 |
| Fourth | Screen det (all)^5,8^ | 352 | 867 | 689 | 984 | 471 | 3,363 |
|  | Screen det (inv)^5^ | 300 | 717 | 593 | 858 | 416 | 2,884 |
|  | % invasive | 85 | 83 | 86 | 87 | 88 | 86 |
|  | Lymph node info^6^ | 293 | 698 | 553 | 799 | 401 | 2,744 |
|  | Lymph node neg^6^ | 217 | 545 | 462 | 671 | 310 | 2,205 |
|  | % neg | 74 | 78 | 84 | 84 | 77 | 80 |
|  | Size info^7^ | 285 | 694 | 529 | 799 | 391 | 2,698 |
|  | Size <10 mm^7^ | 100 | 271 | 218 | 337 | 157 | 1083 |
|  | % <10 mm | 35 | 39 | 41 | 42 | 40 | 40 |

Notes:

1. DKMS 2016, calculated from Table 5A
2. DKMS 2015, Table 6
3. DKMS 2015, Table 7
4. DKMS 2015, Table 8
5. DKMS 2016, Table 6
6. DKMS 2016, Table 7
7. DKMS 2016, Table 8

DKMS 2016, Supplementary tables, Indicator 6, Table 5
