# Supplementary material for: Outcome of breast cancer screening in Denmark
Source: BMC Cancer. 2017 Dec 28;17:897. doi: 10.1186/s12885-017-3929-6 (PMC5745763; doi:10.1186/s12885-017-3929-6)
Supplement: Supplementary file 5 — Number of screened women, screen-detected cancers (invasive + DCIS) interval cancers and women with false positive screens by invitations round and region in screening mammography in Denmark 2008–2015. (DOCX 15 kb) [file 12885_2017_3929_MOESM5_ESM.docx]

Supplementary Table 5. Number of screened women, screen-detected cancers (invasive+DCIS) interval cancers and women with false positive screens by invitations round and region in screening mammography in Denmark 2008-2015.

|  | | Screened women | BC+  DCIS | Inter-val^6^ | BC total | False – positive | Healthy total | Sensit-  ivity | Specificity | 1 –  spec. |
| --- | --- | --- | --- | --- | --- | --- | --- | --- | --- | --- |
| First | North | 57,757^1,2^ | 514^4^ | 103 | 617 | 1,827 | 57,140 | 83.3  (80.1-86.2) | 96.8  (96.7-97.0) | 3.2 |
|  | Central | 114,375^1,2^ | 1,106^4^ | 219 | 1,325 | 2,321 | 113,050 | 83.5  (81.4-85.4) | 97.9  (97.9-98.0) | 2.1 |
|  | South | 107,030^1,2^ | 1,000^4^ | 241 | 1,241 | 1,800 | 105,789 | 80.6  (78.3-82.8) | 98.3  (98.2-98.4) | 1.7 |
|  | Capital | 139,750^1,2^ | 1,286^4^ | 260 | 1,546 | 2,701 | 138,204 | 83.2  (81.2-85.0) | 98.0  (98.0-98.1) | 2.0 |
|  | Zealand | 87,932^1,2^ | 818^4^ | 209 | 1,027 | 1,670 | 86,905 | 79.6  (77.1-82.1) | 98.1  (98.0-98.2) | 1.9 |
|  | DK | 506,844^1,2^ | 4,724^4^ | 1,032 | 5,756 | 10,319 | 501,088 | 82.1  (81.1-83.1) | 97.9  (97.9-98.0) | 2.1 |
| Second | North | 61,141^1,3^ | 358^5^ | 134 | 492 | 1,896 | 60,649 | 72.8  (68.6-76.7) | 96.9  (96.9-97.0) | 3.1 |
|  | Central | 117,455^1,3^ | 773^5^ | 272 | 1,045 | 1,974 | 116,410 | 74.0  (71.2-76.6) | 98.3  (98.2-98.4) | 1.7 |
|  | South | 123,067^1,3^ | 712^5^ | 294 | 1,006 | 2,453 | 122,061 | 70.8  (67.9-73.6) | 98.0  (97.9-98.1) | 2.0 |
|  | Capital | 133,740^1,3^ | 854^5^ | 382 | 1,236 | 2,951 | 132,504 | 69.1  (66.4-71.7) | 97.8  (97.7-97.9) | 2.2 |
|  | Zealand | 46,898^1,3^ | 276^5^ | 125 | 401 | 758 | 46,497 | 68.8  (64.0-73.3) | 98.4  (98.3-98.5) | 1.6 |
|  | DK | 482,301^1,3^ | 2,973^5^ | 1,207 | 4,180 | 10,031 | 478,121 | 71.1  (69.7-72.5) | 97,9  (97.9-97.9) | 2.1 |
| Third | North | 63,490^1,3^ | 381^5^ | 117 | 498 | 1,885 | 62,992 | 76.5  (72.5-80.2) | 97.0  (96.9-97.1) | 3.0 |
|  | Central | 119,179^1,3^ | 776^5^ | 280 | 1,056 | 1,794 | 118,123 | 73.5  (70.7-76.1) | 98.5  (98.4-98.6) | 1.5 |
|  | South | 126,999^1,3^ | 773^5^ | 270 | 1,043 | 2,947 | 125,956 | 74.1  (71.3-76.8) | 97.7  (97.6-97.7) | 2.3 |
|  | Capital | 144,330^1,3^ | 1,109^5^ | 409 | 1,518 | 3,195 | 142,812 | 73.1  (70.8-75.3) | 97.8  (97.8-97.8) | 2.2 |
|  | Zealand | 88,050^1,3^ | 606^5^ | 225 | 831 | 1,446 | 87,219 | 72.9  (69.8-75.9) | 98.3  (98.3-98.4) | 1.7 |
|  | DK | 542,048^1,3^ | 3,645^5^ | 1,301 | 4,946 | 11,222 | 537,102 | 73.7  (72.5-74.9) | 97,9  (97.9-98.0) | 2.1 |

Notes:

1. Only screened women with a screening result
2. DKMS 2015, Table 4
3. DKMS 2016, Table 4
4. DKMS 2015, Supplementary tables, Indicator 4, Table 2
5. DKMS 2016, Supplementary tables, Indicator 6, Table 5
6. DMKS 2016, Table 5A+B
